# Spatial turnover in host-plant availability drives host-associated divergence in a South African leafhopper (*Cephalelus uncinatus*)

**DOI:** 10.1186/s12862-017-0916-0

**Published:** 2017-03-09

**Authors:** Willem J. Augustyn, Bruce Anderson, Jeroen F. van der Merwe, Allan G. Ellis

**Affiliations:** Botany and Zoology Department, University of Stellenbosch, Stellenbosch, South Africa

**Keywords:** Beta diversity, Ecological divergence, Geographic mosaic, Host-shift, Interaction turnover, Local adaptation, Magic traits, Phytophagous insects, Specialization, Speciation

## Abstract

**Background:**

The evolution of reproductive isolation between herbivorous insect populations is often initiated by shifts to novel host-plants, a process that underlies some of the best examples of ecological speciation. However, it is not well understood why host-shifts occur. Arguably the most common hypothesis is that host-shifts occur in response to competition, while a less frequently invoked hypothesis is that herbivores adapt locally to geographic differences in potential host-plant communities. Here we investigate whether geographic variation in host-plant availability is likely to have driven host-shifts in restio leafhoppers. We studied local adaptation of a camouflaged restio leafhopper species, *Cephalelus uncinatus,* to host-plants in the Restionaceae (restios); a family of plants with exceptional diversity in the anomalously species-rich Cape Floristic Region (CFR). To determine whether *C. uncinatus* experiences heterogeneous host communities across its range, we first quantified the degree of geographic overlap between *C. uncinatus* and each of its associated host-plant species. Then we quantified trait divergence (host preference, body size and colour) for three pairs of *C. uncinatus* populations found on different host-plant species differing in their degree of spatial overlap. Spectral reflectance was modelled in bird visual space to investigate whether body colour divergence in *C. uncinatus* corresponds to leaf sheath colour differences between restio species as perceived by potential predators.

**Results:**

We demonstrate that *C. uncinatus* is forced to use different restio species in different regions because of turnover in available host species across its range. Comparisons between geographically separated populations were consistent with local adaptation: restio leafhoppers had preferences for local host-plants over alternative host-plants and matched local plants better in terms of size and colour.

**Conclusions:**

Spatial turnover in host-plant availability has likely facilitated host-shifts in *C. uncinatus*. Spatial turnover in host-plant availability may be an important driver of insect diversification in the CFR and globally.

**Electronic supplementary material:**

The online version of this article (doi:10.1186/s12862-017-0916-0) contains supplementary material, which is available to authorized users.

## Background

**Fig. 1:**
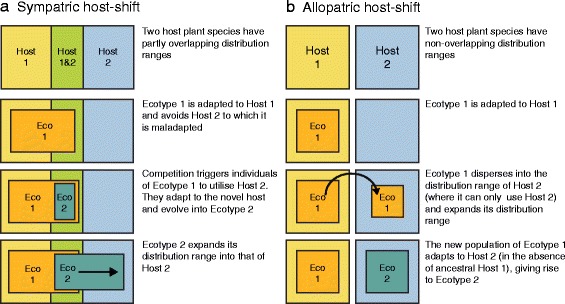
Two models explaining the formation of spatially non-overlapping (or near non-overlapping) ecotypes. Under the sympatric host-shift model (**a**) insects from a population (Eco 1) shift to a novel host-plant (Host 2) and become a new ecotype (Eco 2). Note that the lack of competition [13] on the novel host-plant is often invoked to explain why such shifts occur (see introduction). After the host-shift occurs the new ecotype expands into the distribution range of the novel host (Host 2). Under the allopatric host-shift model (**b**) insects from a population (Eco 1) disperse into a region where its ancestral host-plant is absent (Host 1). It is then forced to use an alternative host-plant (Host 2). It adapts to the novel host-plant and becomes a new ecotype (Eco 2). While the allopatric host-shift model can occur when host-plant distribution ranges are completely non-overlapping or partially non-overlapping, the sympatric host-shift model can only occur when host-plants have partially overlapping distribution ranges. Note that we do not attempt to cover all possible models. See, for example, the more complex specialisation oscillation hypothesis [69]

Speciation by shifting hosts (when populations of parasites adapt to novel hosts [1–3]) is viewed as the primary mode of speciation in herbivorous insects (but see exceptions [4–6]). However, it is not well understood how host-shifts are initiated. One conceptual problem is that, because host-plants can differ markedly in their chemistry [3, 7] and as camouflage backgrounds against predators [8, 9], host shifting insects are likely to be maladapted to their novel hosts. This may render sympatric shifts to a novel host improbable. The problem of maladaptation is mitigated by the tendency of insects to shift between closely related plants representing relatively similar niches (reviewed in [1, 3, 10]). However, this leaves little scope for adaptive divergence as selection gradients will be weaker. Sympatric host-shifts could be facilitated when competition for food on an ancestral host reduces the fitness of insects and drives shifts onto novel hosts [11–14]. By shifting to a novel host, the maladapted insects may still gain in fitness (relative to insects on the ancestral host) if the host shift reduces competition [11–14]. Such a shift is expected to result in disruptive selection and the formation of two discrete host races [1, 14–16]. Once the host-shift has occurred, geographic expansion into the distribution range of the newly colonised host species may ensue, sometimes creating largely allopatric distribution patterns. We refer to this as the sympatric host-shift model (see Fig. 1a). This is the idea that has dominated the literature on host shifts by herbivorous insects [17, 18], despite the fact that herbivorous insects seldom reach densities where food is limiting [19]; perhaps making competition an infrequent driver of sympatric host shifts.

A simpler, but surprisingly, less commonly invoked hypothesis for host shifts is that spatially separated populations of insects experience different host-plants. Local adaptation occurs in allopatry because the different hosts have different chemical or morphological characteristics. This may occur if herbivores expand their ranges into areas with novel host assemblages or if host community composition changes in parts of the existing range of herbivorous insects. Once a population establishes on the novel host-plant, local adaptation can lead to a shift in host preference [20] and lower survival on the ancestral host relative to the novel host [7]. Consequently, specialization on different host-plant species can lead to phenotypically divergent host-ecotypes which become reproductively isolated through assortative host-use and immigrant inviability [21, 22]. We refer to this process as the allopatric host-shift model (Fig. 1b). The pattern produced by the sympatric host-shift model may not be easily distinguished from the allopatric host-shift model if range expansion occurs after a sympatric host-shift (compare Fig. 1a, b). However, the allopatric host-shift model is dependent on high spatial heterogeneity in host-plant availability, whereas the sympatric model is not.

Spatial environmental heterogeneity (for example, in soils, topography, pollinators) is thought to underlie plant diversification in the Cape Floristic Region (CFR) [23], a biodiversity hotspot for plants [24]. In addition, plant diversity has largely accrued through spectacular radiations of a limited number of plant clades [25]. This mode of allopatric plant speciation has resulted in exceptional species turnover between communities, evidenced by the unusually high beta diversity that characterises the CFR [26]. However, these spatially separated plant communities, differing strikingly in species composition, may often comprise phylogenetically closely related species from radiating clades [27–29]. Climatic stability in the region is also thought to have facilitated plant diversification by limiting population extinction and providing ample time for divergence to take place [23] across stable environmental gradients. Together these characteristics of the CFR flora suggest that an allopatric host-shift model of host-ecotype formation is a likely scenario for diversification of the associated insect herbivore lineages. Strong and temporally stable geographic mosaics of host-plant availability are likely to result in divergent selection across spatially separated populations of insect species. Furthermore, the presence of related plant species across spatially separated communities may facilitate the survival of insects dispersing outside the range of their ancestral host-plants. Therefore, spatial heterogeneity is likely to be important in causing allopatric host-shifts in herbivorous insects in the CFR, but it remains to be investigated.

In contrast, fire, a key driver of CFR ecology, may reduce the likelihood of host-shifts in sympatry. Fire disturbance occurs frequently in this region, and the main vegetation type, Fynbos, consists of plants showing a variety of adaptations to survive fires (reviewed in [30]). While some insect species can survive Fynbos fires (reviewed in [30]), many species must recolonise burned vegetation (probably through dispersal from neighbouring unburned vegetation patches), which can take 3 years [31]. Additionally, local plant communities differ stochastically across fire cycles [32]. Divergence between different host-associated insect populations in sympatry may be impeded by these consequences of fire, i.e. regular resetting of population size and insect community composition reducing the likelihood of strong competition, frequent extinction of locally adapting insect populations, and temporally variable selection resulting from both the fire-driven local dispersal/re-colonisation cycle and fire-induced changes in local plant composition [33].

One of the diagnostic plant families within the fynbos is the African Restionaceae (restios hereafter). Restios form a highly diverse, monophyletic plant clade, which comprises about 350 species. They are thought to have originated about 65 million years ago [34], making them one of the oldest clades in the CFR with only 10 species occurring outside the CFR (reviewed in [25]). Restios are generally reed-like in appearance. Their photosynthetic stems (culms) have regular nodes with dried-out leaf sheaths that persist in most species, but drop off in others. The leaf sheaths of restios appear to be mimicked by the morphology of locally host-specific herbivores called restio leafhoppers (Cicadellidae: Cephalelini) [35], the dominant insects on restios [36]. Restio leafhoppers are characterised by small, slender bodies and elongated crowns (with the exception of *Duospina capensis*) resembling the bracts and dried out leaf sheaths of restios [37]. Currently there are 21 described restio leafhopper species from two genera, namely *Cephalelus* and *Duospina* [38]. Evidence suggests that restio leafhoppers did not co-diversify with restios. Instead they diversified much more recently (1–6 MYA) than restios [39], which are approximately 15 times more diverse than restio leafhoppers in terms of the number of described species. Thus, many restio species are not exploited by restio leafhoppers, leaving many unfilled niches onto which restio leafhoppers can potentially radiate.

Our study focuses on *Cephalelus uncinatus*, a broadly distributed restio leafhopper species which completes its entire life cycle on restios. Oviposition occurs on the host-plant and since the eggs have no protective coverings (see e.g. [40]) they are unable to survive fires (WJA, personal observation). *C. uncinatus* uses several genera of restios from the Willdenowieae sub tribe, as well as several species in the genus *Elegia* which belongs to the Restioneae sub tribe [39]. Augustyn et al. [41] demonstrated experimentally that *C. uncinatus* from a single site actively chooses its predominant field host and also survives better on it than on unused restio species. As experiments were performed in the absence of predators, the authors suggest that preference is linked to performance through plant chemistry. However, *C. uncinatus* may also gain protection from predators by choosing restios that serve as good camouflage backgrounds. Previous studies have shown that restio leafhoppers maintain consistent host preference in the presence and absence of interspecific competitors [37], and provide no evidence that intraspecific competition broadens host preference in restio leafhoppers [42]. These studies suggest that competition has not been important in the diversification of restio leafhoppers, leaving geographic mosaics of phenotypically different hosts as a likely explanation for host-shifts.

Across its distribution range, *C. uncinatus* uses different host species, and Prendini [38] suggested that it consists of several ecotypes. He reported consistent, but slight differences in genitalia between populations using *Willdenowia incurvata*, *Mastersiella digitata* and *Elegia nuda* as host-plants (species that differ in culm thickness and leaf sheath colour). Since competition appears to be an unlikely driver of host-shifts in restio leafhoppers [41, 42], we determine whether spatial heterogeneity in plant distributions could have initiated host-shifts in *C. uncinatus*. If so, we expect to find: (1) that host-plant distributions are smaller than the distribution of *C. uncinatus*, thus generating a geographic mosaic in the availability of different hosts across the insect species range, and (2) that host preference, colour and shape of restio leafhoppers are locally adapted to their host-plants across this geographic mosaic. 3) Lastly, only finding evidence for divergence between populations using host-plants with spatially non-overlapping distributions would support an allopatric over a sympatric host-shift model (see Fig. 1).

## Methods

### Distribution overlap between *C. uncinatus* and its hosts

We assessed the spatial heterogeneity of host-plant availability with plant and insect distribution records. We sourced 19 records of *C. uncinatus* from a MSc thesis [43] and 14 from an honours thesis [44]. Through our own opportunistic collecting and standardised sampling we obtained 11 other distribution records [42]. All *C. uncinatus* individuals were either identified by WJA, Davies [43] or Prendini [44]. Restio distribution records were sourced from the leading expert on the Restionaceae, HP Linder (unpublished data). This data set mostly includes records from the Bolus herbarium and fieldwork conducted by HP Linder.

**Fig. 2:**
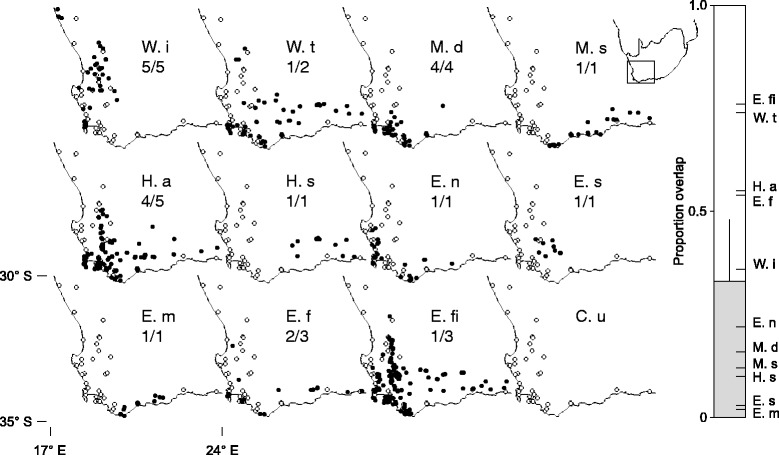
Distribution overlap between *C. uncinatus* and its hosts. Known distribution ranges of *C. uncinatus* (C. u – empty dots) and restios used by it (black dots). These are: *Willdenowia incurvata* (W. i), *Willdenowia teres* (W. t), *Mastersiella digitata* (M. d), *Mastersiella spathulata* (M. s), *Hypodiscus aristatus* (H. a), *Hypodiscus synchroolepis* (H. s), *Elegia nuda* (E. n), *Elegia stokoei* (E. s), *Elegia muirii* (E. m), *Elegia. fistulosa* (E. f) and *Elegia filacea* (E. fi). Fractions show how many times a restio species was used by *C. uncinatus* out of the number of times that the restio species co-occurred with *C. uncinatus* in our dataset (Additional file 1: Table S1)*.* The bar on the right shows the proportion of the range of *C. uncinatus* overlapping with each restio species (indicated with tick marks which correspond to small individual bars in each panel). The grey portion of the bar shows bootstrap determined mean overlap across restio species with an upper 95% confidence interval

Restio species were only considered as hosts and included in the spatial analysis if more than one *C. uncinatus* individual had been captured on them (see Additional file 1: Table S1 for counts). These hosts were *Willdenowia incurvata*, *Willdenowia teres*, *Mastersiella digitata*, *Mastersiella spathulata*, *Hypodiscus aristatus* and *Hypodiscus synchroolepis* from the Willdenowieae sub tribe and *Elegia nuda*, *Elegia stokoei*, *Elegia muirii*, *Elegia fistulosa*, and *Elegia filacea* from the Restioneae (Fig. 2).

For the abovementioned restio species, and *C. uncinatus*, we estimated ranges by calculating convex hulls (polygons based on the outlines of point distributions) around all distribution records using the gConvexhull function in the R package rgeos [45]. We then used the gIntersection function in rgeos to measure the proportion of the *C. uncinatus* range which overlapped with each restio species. Projections and data conversions were performed using the R packages sp [46] and rgdal [47]. By means of a bootstrapping procedure, overlap data were analysed by obtaining a mean and upper 95% CI using the one.boot function in the R package simpleboot [48]. An upper 95% CI lower than 1 indicates that host-plants have ranges that don’t cover the total distribution range of *C. uncinatus*. We reasoned that if none of the host species’ ranges overlap fully with the range of *C. uncinatus*, then by necessity, populations of *C. uncinatus* will vary in their host-use.

### Local adaptation in preference

**Fig. 3:**
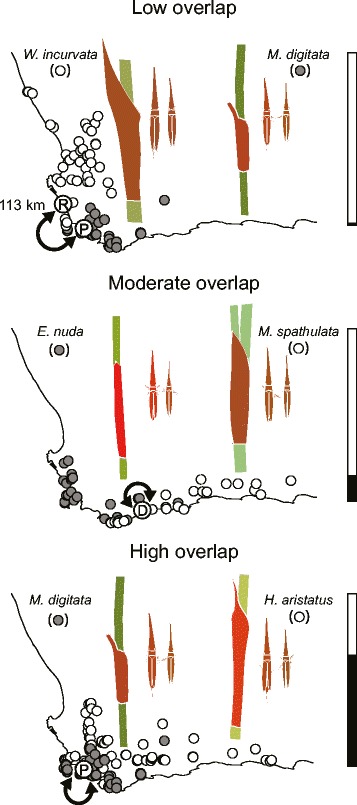
Experimental design. Local adaptation experiments were conducted at three sites: Rondeberg (R), De Hoop (D) and Pringle Bay (P). Three experiments were conducted: a low range overlap comparison (sites were 113 km away from each other) between *C. uncinatus* using *M. digitata* at Pringle Bay and *W. incurvata* at Rondeberg (top panel), a moderate overlap comparison at De Hoop between *C. uncinatus* using *M. spathulata* and *E. nuda* (*middle pane*l), and a high overlap comparison at Pringle Bay between *C. uncinatus* using *M. digitata* and *H. aristatus* (bottom panel). Range overlap between restio species used in each experiment is shown in the *bars* to the right. Restio culms with sheaths are illustrated together with associated *C. uncinatus* ecotypes (smaller males are shown to the right of females). Restio sheath colour and ecotype colour of cartoons were obtained by spectrophotometer and converted to median RGB values for visualisation on a computer screen in human vision. Insects and plants were approximately drawn to scale

We tested for local adaptation in three pairs of restio leafhopper populations. Populations in each pair utilized different host-plants and population pairs differed in their degree of geographic range overlap (range overlap henceforth) (Fig. 3). Range overlap was estimated by measuring the convex hull overlap of restio species pairs (range overlap = proportion of species A range overlapping species B, plus proportion of species B range overlapping species A, all divided by 2). Local adaptation in host preference was investigated between *C. uncinatus* populations using two hosts (*M. digitata* and *W. incurvata)* with low (1%) range overlap (Fig. 3). Low range overlap of host-plants imply that *C. uncinatus* populations will almost always encounter *M. digitata* and *W. incurvata* in isolation of each other. This may facilitate divergent specialization and ecotype formation. At a moderate degree of host range overlap (14.5%), we compared preferences of *C. uncinatus* using *M. spathulata* and *E. nuda* (Fig. 3). Moderate spatial overlap of host-plants suggests that *C. uncinatus* populations will infrequently encounter *M. digitata* and *E. nuda* together, facilitating specialization on different host-plants (likely to a lesser degree than for the low range overlap comparison). At the highest degree of host-plant range overlap, we tested populations occurring on *M. digitata* and *H. aristatus. Mastersiella digitata* and *H. aristatus* have extensively overlapping ranges (64%) (Fig. 3). Therefore, populations of *C. uncinatus* probably frequently encounter *M. digitata* and *H. aristatus* simultaneously so that the lack of geographic separation in these populations is likely to hinder specialization to only one host-plant (unless divergence can occur in sympatry).

The host-plant arrangement at the site level also differed between the three different ecotype pair comparisons. Restio leafhoppers using hosts with low range overlap were collected from either *M. digitata* at Pringle Bay or *W. incurvata* at Rondeberg. These sites are 113 km away from each other, and gene flow should be minimal (Fig. 3). *C. uncinatus* were first collected from *W. incurvata* at Rondeberg on the 28^th^ of October 2013. On the following day *C. uncinatus* was collected from *M. digitata* in Pringle Bay. When the experiment was repeated later, insects were first collected from *M. digitata* on the 19^th^ of November 2013 at Pringle bay and then from *W. incurvata* the following day.

For local adaptation of herbivore populations using host species with moderate overlap, *C. uncinatus* were all collected at De Hoop on stands of either *E. nuda* or *M. spathulata* 800 m away from each other (Fig. 3). It is therefore likely that there is some level of gene flow between populations using different host-plants. Collections were made on the 6^th^ and the 8^th^ of January 2014.

To investigate local adaptation in populations with high host species overlap, insects were collected at Pringle Bay from either *M. digitata* or *H. aristatus* plants that were in both monospecific and mixed stands. It is therefore likely that, at the site level, there is frequent gene flow between populations using either *M. digitata* or *H. aristatus*. Insects were collected on the 6^th^ and the 13^th^ of November 2013*.*

For all experiments, insects were collected by vacuuming them off plants using a modified leaf blower/shredder. Captured insects were placed singly into clean Eppendorf vials, which were then placed in a cooling box. Later, insects were transferred to a fridge at 10 °C until experiments started the next day (approximately 12 h later) or two days later for the low overlap comparison (36 h). Restio culms of the relevant hosts were collected at the insect collecting sites and placed into distilled water to keep them fresh. Once in the laboratory, culms were cut to the length of 135 mm and kept fresh in a fridge at 10 °C.

**Fig. 4:**
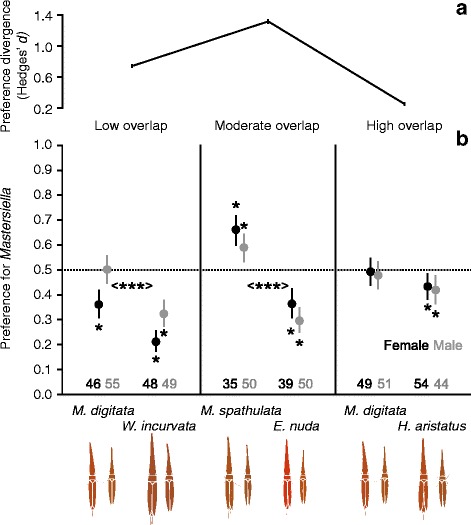
Divergence in host preference. Host preference differences were detected in the low overlap and moderate overlap comparisons, but divergent preference was strongest in the moderate overlap comparison (higher Hedges’ *d* means (variance also shown) indicate stronger host preference divergence in panel **a**). In panel **b** significance in host preference divergence is indicated with < ***>, *P* < 0.001. Names on the x axes correspond to the host-plants insects were collected from. The y axes represent the probability that *Mastersiella* (*M. digitata* or *M. spathulata*) was chosen over the other species, estimated by means of binomial GEEs. GEE estimated means and 95% CI of females (*black*) and males (*grey*) are shown. When bars are above 0.5 it indicates a preference for *Mastersiella*, when below it indicates a preference for the other species (indicated by *). Sample sizes are shown below each mean and 95% CI

In each of the three different host range overlap scenarios, a minimum of 85 individuals of each restio leafhopper population were presented with a choice of both restio species (sample sizes of the ecotypes and separate sexes are shown in Fig. 4). In each case, host preferences by both ecotypes were tested simultaneously. To keep track of the preferences of individual insects (whilst avoiding competitive or sexual interactions), insects were placed alone into 740 ml preserve jars with one cutting of each restio species. Culms were placed in the jar so that they were touching and restio leaf hoppers were placed on the bottom of the jar, equidistant from the two culms. Restio cuttings were kept fresh by placing each into separate 0.6 ml vials filled with water. We prevented fogging of jars by replacing lids with fine gauze, and jars were kept at a constant 25 °C. Insects could easily move over the glass surface of the jars, and from one culm to another. After 12 h, preference of each individual was recorded as the restio species it was sitting on. Repeated observations were made after 15, 18, 21 and finally 24 h.

We analysed host preference data using separate binomial generalised estimating equations (GEE) with log link functions and exchangeable correlation structures for each of the three experiments (correlation structure choice had little effect on models). The response variable was always a binary choice for *Mastersiella* (either *M. digitata* in the low range overlap and high range overlap experiments or *M. spathulata* in the moderate range overlap experiment). Each model tested for the role of the variables time (after start of experiment), sex and population, in determining host choice (whilst accounting for non-independence of repeat measurements from an individual). For each GEE we back transformed and plotted estimates of means and 95% CIs on a scale ranging from 0 to 1. When 95% CIs did not overlap with a preference of 0.5, a preference for one of the compared restio species was inferred. We also calculated standardised effect sizes (Hedges’ *d*) for the population variable using GEE estimated means and standard deviations [49]. All GEEs were implemented in the geepack package [50] in R version 3.2.2.

### Morphological divergence

#### Body size

**Fig. 5:**
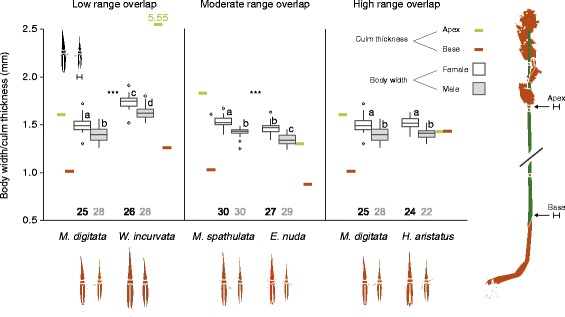
Body size divergence. When populations differed in body width (low and moderate range overlap comparisons) larger bodied populations were associated with restios with thicker culms. Panels correspond to comparisons in Fig. 3. Names on the x axes correspond to the restio species that insects were collected from. Boxplots (with outliers) are shown with hinges corresponding to 25^th^ and 75^th^ percentiles, whiskers correspond to the highest and lowest values within interquartile ranges. Median thickness of restio culm thickness at the apex and base of culms are shown for reference, but culm thickness data were analysed separately (see Additional file 2: Figure S1). Note that the base of *W. incurvata* culms (indicated off-scale as 5.55 mm) were much thicker than those of other restio species. Host effects from two way ANOVAs are shown, significance is indicated by *** (*P <* 0.001). Difference between sexes and ecotypes not sharing letters are significant below *P* = 0.05, as determined by Tukey post hoc tests. Sample sizes are shown below each box

Restio leafhoppers frequently attempt to obscure themselves by circling around to the opposite side of restio culms in relation to perceived predators (a common behaviour of Cicadellidae). The match between restio culm thickness and restio leafhopper body width is likely to play an important role in predator avoidance because thick-bodied restio leafhoppers might not be able to effectively hide behind thin restio culms. As restio species pairs used in the preference experiments differ in culm thickness (thicknesses of the apex and bases of culms were measured with callipers, see Additional file 2: Figure S1), divergence in body width might be expected between *C. uncinatus* population pairs. Specifically, *W. incurvata* has thicker culms than *M. digitata* (low range overlap comparison), *M. spathulata* has thicker culms than *E. nuda* (moderate range overlap comparison), and *H. aristatus* has thicker culms than *M. digitata* (high range overlap comparison) (Additional file 2: Figure S1). Using the specimens from the presentation experiments, we tested whether broader bodied populations are associated with restio species that have thicker culms. To do this, we measured body width and elytron lengths (sample sizes shown in Fig. 5) as measures of body size. For each separate comparison, we then conducted two-way ANOVAs including host-plant and sex as independent variables and body width as the dependant variable. We also performed a similar set of ANOVAs with body width divided by elytron length (stockiness) as a response variable. This was done to investigate whether divergence in thickness has occurred independently from divergence in total body size.

#### Body colour

Body colour is another trait likely to be under divergent selection if the colours of sheaths differ on different restio hosts. More specifically we expect insect body colour to match the colour of host-plant leaf sheaths. This may occur if selection from predation favours restio leafhoppers that colour-match the leaf sheaths of restios on which they occur. Using an Ocean Optics USB4000 spectrometer we recorded spectral colour data of *C. uncinatus* caught on the five different host-plant species used in the reciprocal restio presentation experiments. Before taking colour measurements, the spectrometer was allowed to heat up for approximately 45 min. Thereafter, light and dark calibrations were performed every 10 min. All insects were measured once, while two repeat measures of each restio sheath were taken and averaged*. C. uncinatus* sample sizes are shown in Fig. 5. Restio sheath sample sizes were as follows: 18 individuals of *W. incurvata*, 15 of *M. digitata*, 12 of *H. aristatus*, 10 of *M. spathulata* and 11 of *E. nuda*.

We modelled the spectral data in tetrahedral colour space of potential visual predators (i.e. birds) of restio leafhoppers using the R package PAVO. Specifically, we modelled colour under the average avian VS model [51], which assumes that the birds hunting restio leafhoppers are not very UV (ultraviolet) sensitive. Modelling was conducted assuming D65 standard daylight. Using the colour space model, we tested whether each population colour matches the leaf sheaths of their host better than non-hosts. Specifically, we made the same pairwise comparisons as above (low host overlap, moderate host overlap and high host overlap). For each individual insect, we determined the Δ_S_ in just noticeable differences (JNDs) between itself and the closest restio sheath point within the cluster of sheath measurements of each restio species. A lower Δ_S_ indicates a better colour match between insect and sheath. We used two-way repeated measures ANOVAs including the variables, population origin (i.e. which restio species it is from) and restio species identity. As all individual insects were represented by two measurements (i.e. Δ_S_ relative to host and to non-host), we included individual identity as a random variable. Female and male insects were analysed separately. A significant interaction between population origin and plant species identity would be indicative of local adaptation to sheath colour [52].

## Results

### Distribution overlap between *C. uncinatus* and its hosts

Restios are not homogenously distributed across the distribution range of *C. uncinatus* and there is no restio species that occurs across the entire range of *C. uncinatus* (C. u in Fig. 2)*.* On average, each host species was only present in 33% of the *C. uncinatus* range (48% upper 95% CI). Two broadly distributed species, *W. teres* (Fig. 2, W. i) and *E. filacea* (Fig. 2, E. fi), occur across 74 and 76% of the distribution of *C. uncinatus*, but these are both very rarely used hosts (Additional file 1: Table S1). The majority of species (7 out of 10) occurred across less than 50% of the *C. uncinatus* distribution range. Many of these species only overlapped with a small fraction of the *C. uncinatus* distribution range (as low as 2%). Some species that occurred in only a small part of the *C. uncinatus* distribution range were frequently used by *C. uncinatus*. For example, *M. digitata* (Fig. 2, M. d) occurs in only 16% of *C. uncinatus’* distribution, but is always used when encountered (4/4 times in distribution records). It is also interesting that host species tended to have either North to Southwest or East to Southwest distributions (Fig. 2), so that many restio species overlapped more towards the Southwest. *C. uncinatus* populations towards the Northern or Eastern extremes of its distribution range therefore encounter very different suites of host-plants.

### Divergent preference

Divergent host preferences were found in *C. uncinatus* populations using hosts with low and moderate range overlap but not in populations using hosts with high geographic overlap. Individuals captured on *M. digitata* at Pringle Bay and *W. incurvata* at Rondeberg (from hosts with low range overlap, Fig. 3) had strongly divergent host preferences (Wald = 26.51, *P <* 0.001, Fig. 4a). However, there was still a general preference for *W. incurvata*. Females and males from *W. incurvata* showed a strong preference for this species (95% CI lower than 0.5). Females from *M. digitata* showed a weaker preference for *W. incurvata* than females from *W. incurvata* and males had no preference (95% CI overlapped with 0.5). Generally, males had a stronger preference for *M. digitata* than females (Wald = 15.90, *P <* 0.001, Fig. 4). Time since the start of the experiment did not influence host preference in this model (Wald = 1.70, *P* = 0.192).

Individuals captured on either *M. spathulata* or *E. nuda* at De Hoop (from hosts with moderate range overlap, Fig. 3) also had strongly divergent host preferences (Wald = 67.23, *P <* 0.001, Fig. 3). Both sexes consistently preferred the host-plants on which they were found. Preference differences between the sexes for the moderate host overlap comparison were weak (Wald = 4.11, *P* = 0.043, Fig. 3). Time since the start of the experiment did not have a strong effect on host preference (Wald = 2.87, *P* = 0.090).

Individuals captured on *M. digitata* and *H. aristatus* at Pringle Bay (hosts with high range overlap, Fig. 3) did not have significantly divergent host preferences (Wald = 3.20, *P* = 0.074, Fig. 4). Time since the start of the experiment influenced host preference (Wald = 5.02, *P* = 0.025). Specifically, there was a tendency for insects collected from both plants to prefer *H. aristatus* over time.

### Phenotypic divergence

#### Body size

Divergence in the body dimensions of *C. uncinatus* was found in populations using hosts with both low and moderate range overlap, but not in populations using hosts with high range overlap. Insects from the thinner culmed *M. digitata* at Pringle bay were thinner than those caught from thick culmed *W. incurvata* at Rondeberg (F = 273.29, df = 1, *P <* 0.001), and females tended to be thicker than males (F = 55.31, df = 1, *P <* 0.001) (comparison with populations using hosts with low range overlap, Fig. 5). In this comparison insects from *M. digitata* also tended to be less stocky (body width divided by elytron length) than those on *W. incurvata* (F = 42.235, df = 1, *P <* 0.001), and females tended to be stockier than males (F = 92.185, df = 1, *P <* 0.001) (Additional file 3: Figure S2).

At De Hoop where populations use plants with moderately overlapping distribution ranges (Fig. 5), insects caught from the thicker culmed *M. spathulata* were thicker than those caught from the thin culmed *E. nuda* (F = 52.092, df = 1, *P <* 0.001). Females were also thicker than males (F = 113.163, df = 1, *P <* 0.001). Stockiness, however, did not differ between populations (F = 0.612, df = 1, *P* = 0.436), but females tended to be stockier than males (F = 192.438, *P <* 0.001) (Additional file 3: Figure S2). In other words, unlike for the low overlap comparison, body width divergence in this pair reflected body size divergence alone.

At Pringle bay (where populations used hosts with high range overlap, Fig. 5), there were no notable differences in any body dimensions between insects using different host-plants. Insects caught from *M. digitata* and *H. aristatus* had the same body widths (F = 1.860, df = 1, *P* = 0.176), and like for all the other comparisons, females were thicker than males (F = 65.160, df = 1, *P <* 0.001). Similarly, insects using different host-plants did not differ in stockiness (F = 1.254, df = 1, *P* = 0.265), but females tended to be stockier than males (F = 95.259, df = 1, *P <* 0.001) (Additional file 3: Figure S2). Complete ANOVA results of body width and stockiness are shown in in Additional file 4: Tables S2 and Additional file 5: Table S3.

### Restio sheath colour matching

**Fig. 6:**
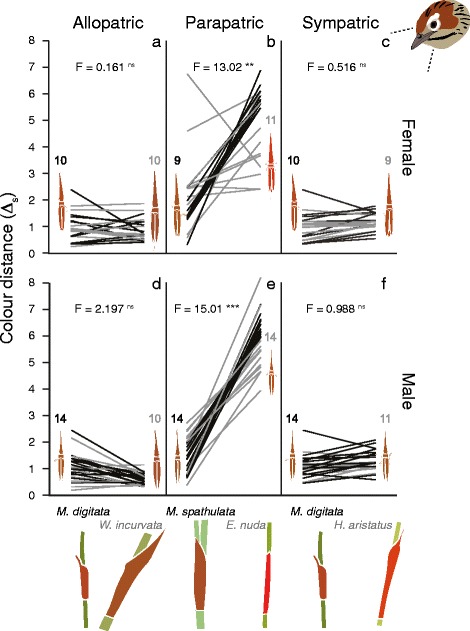
Colour divergence. We detected local adaptation in leaf sheath matching in bird vision in the moderate overlap (**b** and **e**) comparison, but not in the high overlap and (**a** and **d**) and low overlap comparisons (**c** and **f**). Restio species are shown on x axes and the degree of colour matching (Δ_S_ in JNDs) between restio sheaths and insects (in bird vision) are shown on the y axes. A lower Δ_S_ is indicative of a closer match between an insect and a leaf sheath (0 would be a perfect match). Each line connects two measurements on an individual insect. *Black* lines indicate populations associated with host-plants on the left of each panel (always *Mastersiella* spp.) and those that are grey represent populations associated with species on the right (*W. incurvata*, *E. nuda* or *H. aristatus*). Interaction effects from repeated measures ANOVAs are shown on each graph. Strong interaction effects are indicative of local adaptation (*** indicates *P <* 0.001, ** *P <* 0.01, and ns *P >* 0.05). Sample sizes associated with each population are shown above leafhopper cartoons. Restio sheath colour and ecotype colour of cartoons were obtained by spectrophotometer and converted to median RGB values for visualisation in human vision

We observed clear evidence (i.e. significant interaction effects) for local adaptation in leaf sheath colour matching in the comparison where populations were using host-plants with moderate range overlap, but not in the comparisons where host-plants exhibited low and high range overlap (Fig. 6). In the comparison where populations were using host-plants with moderate range overlap, both females (Fig. 6b) and males (Fig. 6e) had divergent body colour. Interestingly, both ecotypes in this comparison matched the leaf sheaths of *M. spathulata* better than those of *E. nuda* (Fig. 6b and e), but the *E. nuda* ecotype consistently matched *E. nuda* sheaths more closely than the *M. spathulata* ecotypes. In the comparison where populations were using host-plants with high range overlap we found no evidence for local adaptation, and *M. digitata* leaf sheaths were matched better than *H. aristatus* leaf sheaths (Fig. 6c and f, see Additional file 6: Table S4). All ANOVA results on leaf sheath matching are shown in Additional file 6: Table S4.

## Discussion

Unlike most taxa, where allopatric over sympatric divergence is typically invoked as the most important pathway to speciation (reviewed in [53]); a great deal of emphasis has been placed on sympatric speciation in herbivorous insects [17, 18]. This manuscript clearly demonstrates that the geographic heterogeneity required for allopatric divergence is ubiquitous in this system. Furthermore, it also demonstrates instances of insect local adaptation to different allopatric host-plants. We suggest that like most other taxa, allopatry may be the dominant pathway to ecological speciation in herbivorous insects and that the role of sympatric divergence in herbivorous insects may have been overplayed in the literature. Below, we evaluate the evidence in support of geographic host-plant mosaics and ecotype formation in allopatry as assessed through host preference and morphological divergence.

### Spatial variation

Here we provide rare empirical evidence suggesting that host-plant mosaics underlie ecologically driven divergence of insects in the CFR. Seven out of the ten known host-plant species, potentially acting as different selective environments (*sensu* [3]), have distribution ranges that overlap with less than 50% of the distribution range of *C. uncinatus*. A similar finding was reported by Kemp et al. [54] who found that spatially separated herbivorous insect populations (and species) on restios often use a different suite of host-plants because of spatial turnover of host-plants. This pattern is mirrored by herbivorous insects in the tropics [55], suggesting that it may be common for spatially separated herbivorous insect populations of the same species to experience divergent selection as a result of plant species turnover. While there is also evidence that host-shifts can occur in sympatry [1, 56], host-shifts resulting from geographic turnover of host-plants may be commonplace. Allopatric shift models are also thought to be important in initiating ecological speciation in plants [57–59] and most other taxa [60]. We suggest that divergence of herbivorous insects may not be different from other taxa in terms of the strong role played by allopatry.

### Divergence in host preference

Divergence in host preference plays an important role in insect speciation because it can directly result in assortative mating [61]. For this reason, host preference can be viewed as a “magic trait” promoting reproductive isolation (*sensu* [62]). However, no studies to our knowledge have explicitly shown that female and male insects prefer the same host-plants (in the context of divergence), which is essential for divergent host preference to result in assortative mating (implied by [63]). In our study, females and males differed slightly in host preference. Nonetheless, different sexes of the same ecotype always had more similar host preferences than different sexes from different ecotypes (see Fig. 4). Thus, considering that *C. uncinatus* mates on its host-plants (WJA, personal observation), divergent host preference can result in assortative mating. Host preference divergence was strongest in the comparison where populations used hosts exhibiting moderate range overlap, and absent in the comparison where populations were using host-plants with high range overlap (Fig. 4). This partly suggests that, like in other systems, divergent host preference can be maintained in the presence of moderate gene flow [20], or that assortative mating via divergent host preference is preventing maladaptive gene flow [64]. Therefore, even if host preference is developmentally plastic, preferences for different host-plants should decrease gene flow, thereby facilitating adaptive divergence in heritable traits.

### Morphological divergence

In this study, we measured traits that are likely under selection from predation. Similar to the preference experiments we cannot rule out the possibility of developmental plasticity or that trait values resulted from non-adaptive processes. Nonetheless, consistent with the findings from preference experiments, we only detected divergent body width in population pairs using host-plants with low and moderate range overlap, suggesting that divergence is adaptive and not due to developmental plasticity. The population pair that used host-plants exhibiting low range overlap also differed in body width relative to body size, while no such scaling difference was found between the moderate overlap population pair. Additionally, larger individuals were always found on plants with thicker culms, suggesting that natural selection has driven body size divergence. Divergent body size could have resulted from predators selecting for smaller (or thinner) insects on thinner culms, allowing them to hide behind culms. Alternatively, large bodied restio leafhoppers may not be able to hold on to thin culms in strong winds and vice versa. Besides camouflage there might, therefore, be a fitness cost to poor mechanical fit (reviewed in [65]), and therefore divergent selection on body size and width.

Regardless of what drives body size, our findings suggest that there is divergence in body size, which is often regarded as a classic magic trait because of its direct involvement in mate choice [62]. Body size in Bahamas mosquitofish, for example, is involved in mate selection whilst simultaneously under divergent selection from predation [66]. This is thought to play an important role in early stage reproductive isolation between populations of this species [66]. While we are not aware of any reported cases of divergent body size causing sexual isolation between populations of herbivorous insect species, male insects often choose mates based on their body size [67]. Thus, body size in *C. uncinatus* might be an important trait involved in assortative mating and a promoter of rapid reproductive isolation. Our findings, therefore, warrant further investigation into body size divergence and sexual isolation in *C. uncinatus*.

Local adaptation in body colour partly reflected preference experiments and body width/size divergence. In bird vision, only *C. uncinatus* from populations using host-plants with moderate range overlap showed evidence for local adaptation in leaf sheath colour matching. No such evidence was found for population pairs using host-plants with low and high distribution overlap (Fig. 6 left and right panels). Nonetheless, both populations using host-plants with moderate range overlap (Fig. 3 middle panel) matched *M. spathulata* leaf sheaths best (despite a significant host x population interaction effect which is indicative of local adaptation, Fig. 6 middle panels). In addition, *E. nuda* females showed more between individual colour variation than females from *M. digitata* (some *E. nuda* females are dark red while others are browner) (Fig. 6 middle panels). One explanation for this apparent maladaptation [68] is that gene flow is occurring from the *M. spathulata* to the *E. nuda* ecotype. Nevertheless, some degree of divergence is maintained despite the possibility of gene flow between the *M. spathulata* and *E. nuda* populations. Although reduced fitness in alternative environments is not directly related to assortative mating, it can indirectly increase reproductive isolation through immigrant inviability [22]. This process only requires that maladapted immigrants have reduced survivorship prior to mating, which as a by-product results in assortative mating [22, 62]. Considering that predatory birds should be better at detecting immigrants in the moderate distribution range overlap comparison (especially females), immigrant inviability may cause reproductive isolation.

## Conclusion

Multiple axes of phenotypic divergence (divergent habitat preference, body size and body colour) driven by host-shifts might facilitate reproductive isolation between *C. uncinatus* ecotypes. Another divergent selection pressure that might cause immigrant inviability is plant physiology. We previously showed that *C. uncinatus* adults using *H. aristatus* have reduced survival on *Elegia filacea*, a lower ranking host species [41]. It is therefore conceivable that reproductive isolation is, in addition to e.g. predation, maintained by immigrant inviability through physiological trade-offs. Closely related restio leafhopper species tend to use closely related restio species [39] suggesting that shifts occur between physiologically and morphologically similar species. Therefore, populations may be able to establish on novel host-plants without escape from competition playing an important role in facilitating the shift.

The presence of restio leafhopper ecotypes in allopatry and a geographic mosaic of host-plants makes it very likely that host-shifts occur in allopatry when herbivores encounter novel host communities. While this manuscript does not provide strong evidence to refute the alternative sympatric pathway of host-shifting; previous manuscripts [41, 42], find no evidence for interspecific [42] and intraspecific [41] competition in restio leafhoppers, a key element of sympatric host shifting [12–14]. Consequently, we suggest that host-shifts in this system are likely driven by restio leafhoppers adapting allopatrically to novel host-plants. This may occur if restio leafhoppers expand their range into sites with different suites of restios (Fig. 1b). One additional line of evidence supports this pathway; divergent host preference and morphology is evident in population pairs exploiting hosts with little range overlap (i.e. in allopatry), and absent in the high range overlap (i.e. in sympatry) comparison. This line of evidence would be much stronger if experiments were replicated across multiple allopatric and sympatric host-plant pairs [20], and we suggest that it could be a fertile area for future study, in this and other insect herbivore systems.

## Additional files

Additional file 1: Table S1. Hosts with more than one *C. uncinatus* host-use record. The second column shows host-use counts (i.e. number of insects caught per host) and the third shows how many times a host was used when it was present in the sampled community. (DOC 32 kb)
Additional file 2: Figure S1. Differences in culm thickness between restio species used by *C. uncinatus* in pairwise comparisons. By means of callipers measurements were taken at the apex and base (see illustration on the right) of plants in the field where insects were collected for preference experiments. Names on the x axes correspond to the restio species that insects were collected from. Boxplots (with outliers) are shown with hinges corresponding to 25^th^ and 75^th^ percentiles, whiskers correspond to the highest and lowest values within interquartile ranges. Note that the base of *W. incurvata* culms (grey insert) were much thicker than those of other restio species. Data were analysed separately in each pairwise comparison with two-way ANOVAs including restio species and position of measurement (apex or base) as independent variables and culm thickness as the dependant variable. F statistics and P values for each comparison are shown. Difference between restio species and position on culm not sharing letters are significant below *P* = 0.05, as determined by Tukey post hoc tests. Sample sizes are shown below boxes. (DOC 203 kb)
Additional file 3: Figure S2. Comparison of width relative to elytra length (stockiness) between *C. uncinatus* populations. Panels correspond to comparisons in Fig. 2 (main text). Names on the x axes correspond to the host-plants that insects were collected from. Boxplots (with outliers) are shown with hinges corresponding to 25^**th**^ and 75^**th**^ percentiles, whiskers correspond to the highest and lowest values within interquartile ranges. Host effects from two way ANOVAs are shown, significance is indicated by *** (*P <* 0.001). Sexes and ecotypes not sharing letters are significantly different, as determined by post hoc tests. Sample sizes are shown below each box. (DOC 118 kb)
Additional file 4: Table S2. ANOVA testing for the effect of host-plant origin, sex and the interaction between host-plant origin and sex on body width. (DOC 31 kb)
Additional file 5: Table S3. ANOVA testing for the effect of host-plant origin, sex and the interaction between host-plant origin and sex on stockiness. (DOC 31 kb)
Additional file 6: Table S4. Repeated measures ANOVAs testing for the effect of host-plant origin, plant identity and the interaction between host-plant origin and plant identity on sheath colour matching. (DOC 36 kb)

## Abbreviations

CFR: Cape Floristic Region
GEE: Generalised estimating equation
JND: Just noticeable differences
MYA: Million years ago
UV: Ultraviolet
