# Supplementary material for: Spatial turnover in host-plant availability drives host-associated divergence in a South African leafhopper (Cephalelus uncinatus)
Source: BMC Evol Biol. 2017 Mar 9;17:72. doi: 10.1186/s12862-017-0916-0 (PMC5343415; doi:10.1186/s12862-017-0916-0)
Supplement: Additional file 1: Table S1. — Hosts with more than one C. uncinatus host-use record. The second column shows host-use counts (i.e. number of insects caught per host) and the third shows how many times a host was used when it was present in the sampled community. (DOC 32 kb) [file 12862_2017_916_MOESM1_ESM.doc]

Table S1: Hosts with more than one *C. uncinatus* host-use record. The second column shows host-use counts (i.e. number of insects caught per host) and the third shows how many times a host was used when it was present in the sampled community.

| Host species | Number of individuals | Used/Available |
| --- | --- | --- |
| *Willdenowia incurvata* | 54 | 5/5 |
| *Willdenowia teres* | 2 | 1/2 |
| *Mastersiella digitata* | 99 | 4/4 |
| *Mastersiella spathulata* | 22 | 1/1 |
| *Hypodiscus aristatus* | 23 | 4/5 |
| *Hypodiscus synchroolepis* | 9 | 1/1 |
| *Elegia nuda* | 7 | 1/1 |
| *Elegia stokoei* | 6 | 1/1 |
| *Elegia muirii* | 4 | 1/1 |
| *Elegia fistulosa* | 11 | 2/3 |
| *Elegia filacea* | 2 | 1/3 |
