# Supplementary material for: Spatial turnover in host-plant availability drives host-associated divergence in a South African leafhopper (Cephalelus uncinatus)
Source: BMC Evol Biol. 2017 Mar 9;17:72. doi: 10.1186/s12862-017-0916-0 (PMC5343415; doi:10.1186/s12862-017-0916-0)
Supplement: Additional file 2: Figure S1. — Differences in culm thickness between restio species used by C. uncinatus in pairwise comparisons. By means of callipers measurements were taken at the apex and base (see illustration on the right) of plants in the field where insects were collected for preference experiments. Names on the x axes correspond to the restio species that insects were collected from. Boxplots (with outliers) are shown with hinges corresponding to 25th and 75th percentiles, whiskers correspond to the highest and lowest values within interquartile ranges. Note that the base of W. incurvata culms (grey insert) were much thicker than those of other restio species. Data were analysed separately in each pairwise comparison with two-way ANOVAs including restio species and position of measurement (apex or base) as independent variables and culm thickness as the dependant variable. F statistics and P values for each comparison are shown. Difference between restio species and position on culm not sharing letters are significant below P = 0.05, as determined by Tukey post hoc tests. Sample sizes are shown below boxes. (DOC 203 kb) [file 12862_2017_916_MOESM2_ESM.doc]

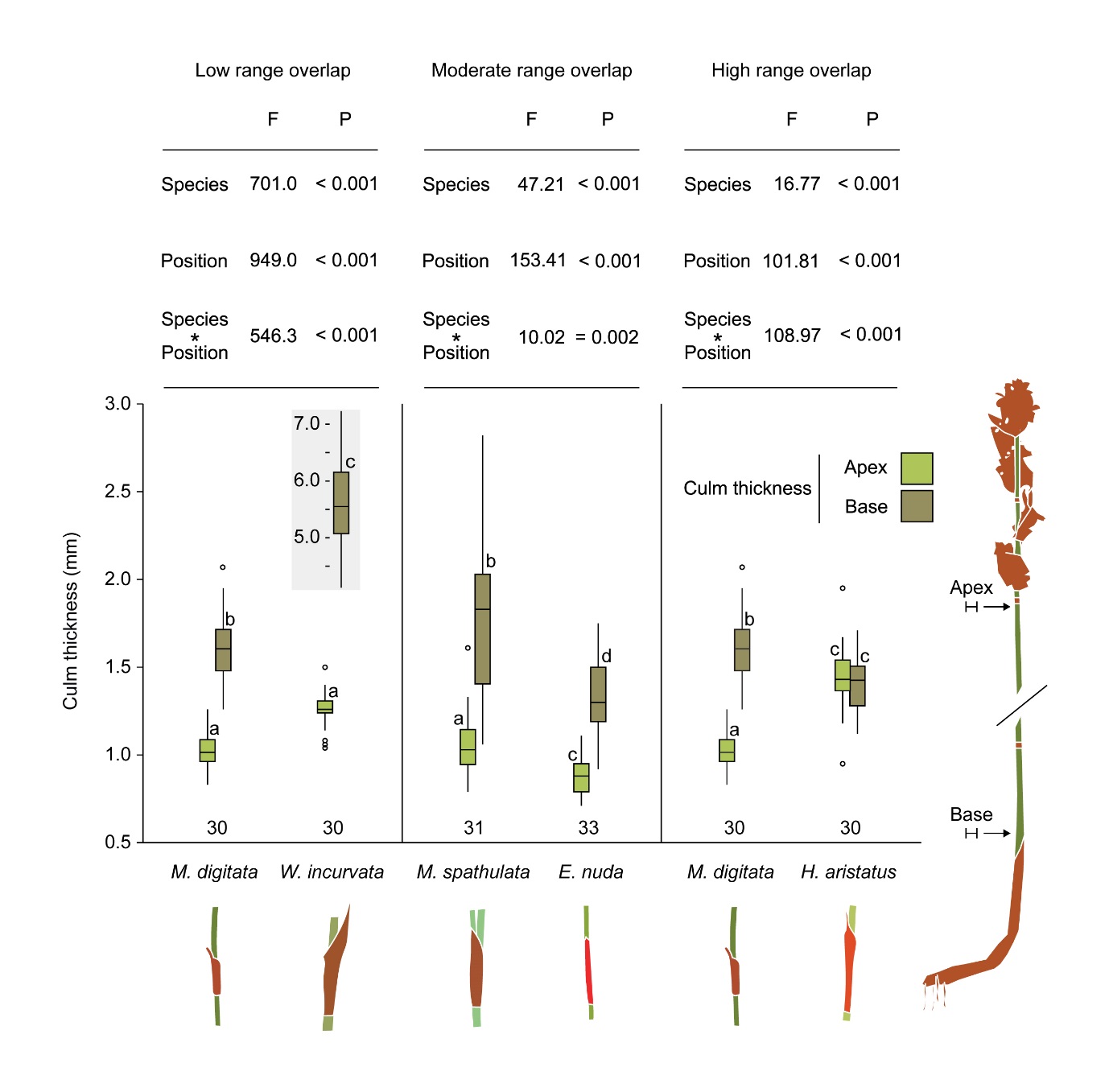


Figure S1: Differences in culm thickness between restio species used by *C. uncinatus* in pairwise comparisons. By means of callipers measurements were taken at the apex and base (see illustration on the right) of plants in the field where insects were collected for preference experiments. Names on the x axes correspond to the restio species that insects were collected from. Boxplots (with outliers) are shown with hinges corresponding to 25^th^ and 75^th^ percentiles, whiskers correspond to the highest and lowest values within interquartile ranges. Note that the base of *W. incurvata* culms (grey insert) were much thicker than those of other restio species. Data were analysed separately in each pairwise comparison with two-way ANOVAs including restio species and position of measurement (apex or base) as independent variables and culm thickness as the dependant variable. F statistics and P values for each comparison are shown. Difference between restio species and position on culm not sharing letters are significant below *P* = 0.05, as determined by Tukey post hoc tests. Sample sizes are shown below boxes.
