# Supplementary material for: Spatial turnover in host-plant availability drives host-associated divergence in a South African leafhopper (Cephalelus uncinatus)
Source: BMC Evol Biol. 2017 Mar 9;17:72. doi: 10.1186/s12862-017-0916-0 (PMC5343415; doi:10.1186/s12862-017-0916-0)
Supplement: Additional file 3: Figure S2. — Comparison of width relative to elytra length (stockiness) between C. uncinatus populations. Panels correspond to comparisons in Fig. 2 (main text). Names on the x axes correspond to the host-plants that insects were collected from. Boxplots (with outliers) are shown with hinges corresponding to 25th and 75th percentiles, whiskers correspond to the highest and lowest values within interquartile ranges. Host effects from two way ANOVAs are shown, significance is indicated by *** (P < 0.001). Sexes and ecotypes not sharing letters are significantly different, as determined by post hoc tests. Sample sizes are shown below each box. (DOC 118 kb) [file 12862_2017_916_MOESM3_ESM.doc]

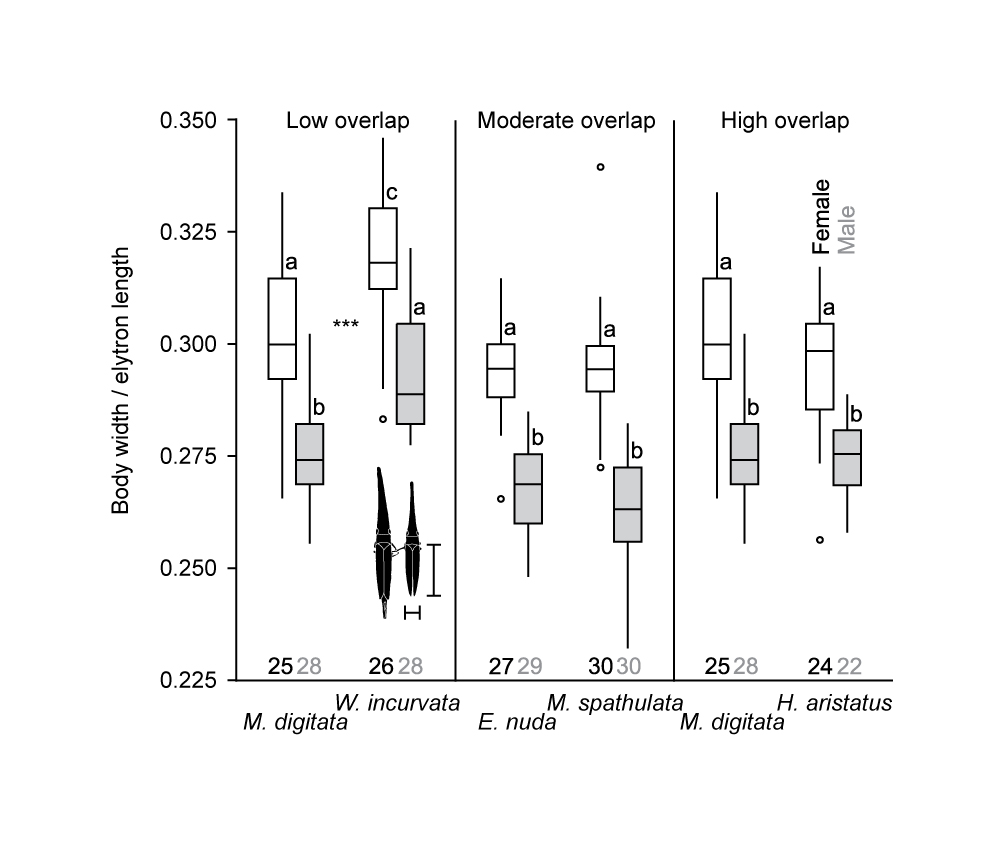
Figure S2: Comparison of width relative to elytra length (stockiness) between *C. uncinatus* populations. Panels correspond to comparisons in Figure 2 (main text). Names on the x axes correspond to the host-plants that insects were collected from. Boxplots (with outliers) are shown with hinges corresponding to 25**^th^** and 75**^th^** percentiles, whiskers correspond to the highest and lowest values within interquartile ranges. Host effects from two way ANOVAs are shown, significance is indicated by *** (*P <* 0.001). Sexes and ecotypes not sharing letters are significantly different, as determined by post hoc tests. Sample sizes are shown below each box.
