# Supplementary material for: Spatial turnover in host-plant availability drives host-associated divergence in a South African leafhopper (Cephalelus uncinatus)
Source: BMC Evol Biol. 2017 Mar 9;17:72. doi: 10.1186/s12862-017-0916-0 (PMC5343415; doi:10.1186/s12862-017-0916-0)
Supplement: Additional file 4: Table S2. — ANOVA testing for the effect of host-plant origin, sex and the interaction between host-plant origin and sex on body width. (DOC 31 kb) [file 12862_2017_916_MOESM4_ESM.doc]

Table S2: ANOVA testing for the effect of host-plant origin, sex and the interaction between host-plant origin and sex on body width.

| Comparison | Factor | df | F | *P* |
| --- | --- | --- | --- | --- |
| Low overlap | Host | 1 | 273.29 | < 0.001 |
|  | Sex | 1 | 55.31 | < 0.001 |
|  | Host * Sex | 1 | 0 | 0.99 |
| Moderate overlap | Host | 1 | 52.092 | < 0.001 |
|  | Sex | 1 | 113.163 | < 0.001 |
|  | Host * Sex | 1 | 0.244 | 0.623 |
| High overlap | Host | 1 | 1.860 | 0.176 |
|  | Sex | 1 | 65.160 | < 0.001 |
|  | Host * Sex | 1 | 0.003 | 0.958 |
