# Supplementary material for: Spatial turnover in host-plant availability drives host-associated divergence in a South African leafhopper (Cephalelus uncinatus)
Source: BMC Evol Biol. 2017 Mar 9;17:72. doi: 10.1186/s12862-017-0916-0 (PMC5343415; doi:10.1186/s12862-017-0916-0)
Supplement: Additional file 5: Table S3. — ANOVA testing for the effect of host-plant origin, sex and the interaction between host-plant origin and sex on stockiness. (DOC 31 kb) [file 12862_2017_916_MOESM5_ESM.doc]

Table S3: ANOVA testing for the effect of host-plant origin, sex and the interaction between host-plant origin and sex on stockiness.

| Comparison | Factor | df | F | *P* |
| --- | --- | --- | --- | --- |
| Low overlap | Host | 1 | 42.235 | < 0.001 |
|  | Sex | 1 | 92.185 | < 0.001 |
|  | Host * Sex | 1 | 0.154 | 0.695 |
| Moderate overlap | Host | 1 | 0.612 | 0.436 |
|  | Sex | 1 | 192.438 | < 0.001 |
|  | Host * Sex | 1 | 1.092 | 0.298 |
| High overlap | Host | 1 | 1.254 | 0.176 |
|  | Sex | 1 | 95.259 | < 0.001 |
|  | Host * Sex | 1 | 1.528 | 0.219 |
