# Supplementary material for: Spatial turnover in host-plant availability drives host-associated divergence in a South African leafhopper (Cephalelus uncinatus)
Source: BMC Evol Biol. 2017 Mar 9;17:72. doi: 10.1186/s12862-017-0916-0 (PMC5343415; doi:10.1186/s12862-017-0916-0)
Supplement: Additional file 6: Table S4. — Repeated measures ANOVAs testing for the effect of host-plant origin, plant identity and the interaction between host-plant origin and plant identity on sheath colour matching. (DOC 36 kb) [file 12862_2017_916_MOESM6_ESM.doc]

Table S4: Repeated measures ANOVAs testing for the effect of host-plant origin, plant identity and the interaction between host-plant origin and plant identity on sheath colour matching.

| Comparison | Factor | df | F | P |
| --- | --- | --- | --- | --- |
| Low overlap | Origin | 1 | 0.008 | 0.931 |
| Females | Plant | 1 | 0.607 | 0.446 |
|  | Origin * Plant | 1 | 0.161 | 0.693 |
| Low overlap | Origin | 1 | 0.64 | 0.432 |
| Males | Plant | 1 | 21.101 | < 0.001 |
|  | Origin * Plant | 1 | 2.197 | 0.152 |
| Moderate overlap | Origin | 1 | 0.824 | 0.376 |
| Females | Plant | 1 | 46.39 | < 0.001 |
|  | Origin * Plant | 1 | 13.02 | 0.002 |
| Moderate overlap | Origin | 1 | 0.216 | 0.646 |
| Males | Plant | 1 | 522.96 | < 0.001 |
|  | Origin * Plant | 1 | 15.01 | < 0.001 |
| High overlap | Origin | 1 | 0.113 | 0.741 |
| Females | Plant | 1 | 6.075 | 0.023 |
|  | Origin * Plant | 1 | 0.516 | 0.482 |
| Low overlap | Origin | 1 | 2.368 | 0.137 |
| Males | Plant | 1 | 9.855 | 0.005 |
|  | Origin * Plant | 1 | 0.988 | 0.3307 |
